# Psychological and behavioral experiences of individuals investing in cryptocurrency in an uncertain environment: a phenomenological approach

**DOI:** 10.3389/fpsyg.2026.1775360

**Published:** 2026-10-20

**Authors:** Şafak Sönmez Soydaş, Mohamed Djafar Henni, Abdulkadir Barut

**Affiliations:** 1 Department of Property Protection and Security, İrfan Can Köse Vocational School, Gümüşhane University, Gümüşhane, Türkiye; 2 Islamic University of Madinah, Medina, Saudi Arabia; 3 Department of Accounting and Taxation, Siverek Vocational School, Harran University, Şanlıurfa, Türkiye; 4 Department of Finance and Banking, Recep Tayyip Erdogan University, Rize, Türkiye; 5 ARUCAD Research Centre, Arkin University of Creative Arts and Design (ARUCAD), Kyrenia, Türkiye

**Keywords:** behavioral finance, coping strategies, cryptocurrency investment, emotion regulation, investor behavior, phenomenology, social identity

## Abstract

**Introduction:**

This study explores, from a phenomenological perspective, how cryptocurrency investors experience emotional, cognitive, and social processes in an uncertain environment and how these experiences influence decision-making, risk management, and identity perception.

**Methods:**

This qualitative phenomenological study involved semi-structured, in-depth interviews with 19 participants selected through criterion sampling. With the participants’ consent, all interviews were audio-recorded, transcribed verbatim, and analyzed thematically using Colaizzi’s approach. The study was reported in accordance with the COREQ framework.

**Results:**

The analysis revealed five main themes: (1) emotional and cognitive processes changing over time, including a transition from intuitive responses to analytical strategies and learning from losses; (2) the influence of social interactions on investment motivation, including guidance from family, friends, and social media and the reassessment of trust dynamics; (3) internal emotional responses to uncertainty, such as panic selling and poor timing, alongside increasing self-regulation and rule-based decision-making; (4) the role of social support in strengthening motivation and coping with stress, as well as feelings of loneliness and burden in its absence; and (5) identity dilemmas and self-awareness, reflected in tensions involving the need for control and burnout, the strengthening of investor identity, and the reorganisation of life priorities.

**Discussion:**

Cryptocurrency investment is not merely a financial activity but a multilayered experience involving emotion regulation, the filtering of social influences, and identity construction. The findings support the development of training programmes integrating behavioral finance, emotion management, and stress-coping strategies. Social media literacy, rule-based trading tools, peer-support channels, and wellbeing-oriented guidance may also help investors manage uncertainty and its psychological consequences.

## Introduction

This study approaches cryptocurrency investment not only as an economic activity but as a socio-psychological process shaped by the dynamic interaction of emotional experiences, cognitive evaluations, social relationships, and behavioral adaptation. Rather than treating investors as isolated decision-makers, the study conceptualizes cryptocurrency investment as a lived experience in which emotions, social interactions, coping efforts, and identity construction continuously influence one another under conditions of uncertainty. The cryptocurrency market has experienced unprecedented growth by 2025, reaching a total market value of $3.46 trillion (Coingecko, 2025). Approximately 60.7% of this value belongs to Bitcoin, reflecting its dominant position among digital assets and the confidence it inspires among investors. Alongside Bitcoin, major crypto assets such as Ethereum, Tether, XRP, and Solana constitute a significant portion of the market. This rapid expansion, supported by factors such as institutional investor interest, spot ETF approvals, and integration into traditional financial systems (The Economic Times, 2025), has not only transformed financial markets but has also created a socio-psychologically complex environment in which emotional, cognitive, and social processes continuously shape investment decision-making.

Cryptocurrencies are defined as decentralized digital currencies operating through blockchain technology, enabling individuals to store and transfer assets through distributed digital ledgers (Corbet et al., 2019). This decentralized structure allows continuous trading without intermediaries, increasing accessibility and participation (Delfabbro et al., 2021a). Investors engage in cryptocurrency trading through online platforms and hybrid financial instruments such as ETFs and derivatives (Hackethal et al., 2022). However, beyond their technical and financial characteristics, cryptocurrency markets represent socio-psychological environments in which investment behavior emerges through the interaction of emotional responses, cognitive evaluations, social influence, and identity-related processes.

Unlike traditional financial markets, cryptocurrency markets are characterized by extreme volatility, regulatory ambiguity, information asymmetry, and exposure to misleading signals. Such conditions create highly uncertain environments that intensify emotional responses, increase perceived risk, and challenge individuals’ sense of control. In these contexts, decision-making processes are not solely driven by rational evaluation but are significantly influenced by cognitive biases and affective states (Kahneman and Tversky, 1979; Bland et al., 2024). Uncertainty may evoke anxiety, a need for control, loss of confidence, concerns about the future, and long-term psychological strain, while also increasing susceptibility to externally driven cues and internally driven emotional cycles (Papadamou et al., 2023). In response to this socio-psychologically demanding environment, investors develop a range of cognitive, emotional, and social strategies to cope with uncertainty. One of the most prominent cognitive tendencies observed in such contexts is the illusion of control (Langer, 1975), reflecting individuals’ belief that they can influence outcomes despite objective uncertainty. This tendency may lead investors to overestimate their decision-making accuracy and underestimate potential losses (Ricciardi and Simon, 2000). Similarly, overconfidence bias represents a cognitive distortion in which individuals place excessive trust in their knowledge and judgments, particularly under conditions of uncertainty (Sudzina et al., 2021). These biases illustrate how investment behavior is shaped by underlying psychological mechanisms rather than purely rational calculations.

Another important psychological tendency is confirmation bias (Nickerson, 1998), which reflects individuals’ inclination to seek and interpret information in ways that confirm their existing beliefs. In digital environments, this tendency is amplified by exposure to selective and often misleading information, leading to distorted decision-making processes (Garcia and Schweitzer, 2015). Additionally, status quo bias (Samuelson and Zeckhauser, 1988) reflects a preference for maintaining existing decisions even in the presence of contradictory evidence, highlighting the role of psychological inertia in financial behavior. From a behavioral finance perspective, these patterns demonstrate that investment decisions are influenced by a combination of emotional, cognitive, and social processes (Ricciardi and Simon, 2000). In particular, biases such as herding behavior, overoptimism, loss aversion, and framing effects shape how individuals perceive risk and make investment decisions (Kahneman and Tversky, 1979; Samuelson and Zeckhauser, 1988). Herd mentality is widely observed, particularly on community-based platforms such as social media, online forums, and investor networks. In these environments, individuals may disregard their own evaluations and instead follow the decisions and recommendations of others (Bikhchandani et al., 1992). This tendency demonstrates how investors often attempt to reduce uncertainty through collective judgments rather than independent analysis. Papadamou et al. (2023) argue that discussions shared on platforms such as Reddit provide strong indicators of investor sentiment and behavioral tendencies. It is particularly evident that the investment decisions of groups gathering around cryptocurrency discussions on social media are shaped not only by market data but also by the intensity and emotional nature of social interactions (Garcia and Schweitzer, 2015).

The framing effect further illustrates how individuals’ risk perceptions and decisions vary depending on how information is presented (Tversky and Kahneman, 1981). Identical information may lead to different decisions when framed as potential gains or losses, highlighting the role of cognitive interpretation in financial behavior. Moreover, periods of high volatility may induce heightened emotional arousal, which can influence decision-making through neuropsychological mechanisms such as rewards sensitivity, dopamine-driven responses, and risk perception (Jain et al., 2025). These conditions may create a form of “financial arousal,” where decisions are driven more by emotional responses than by analytical reasoning.

For some investors, participation in cryptocurrency markets extends beyond financial gain and reflects a deeper psychological process related to self-regulation and future-oriented thinking. Investment decisions may represent attempts to manage uncertainty, reduce existential anxiety, and establish a sense of control over an unpredictable environment (Perepelitsa, 2021). In this sense, cryptocurrency investment can be conceptualized as a psychologically meaningful activity shaped by individuals’ emotional needs, behavioral adaptation, and cognitive interpretations. Following the digital transformation of financial systems, cryptocurrency markets have emerged as a critical context for understanding investor behavior. Existing research demonstrates that investment decisions are influenced not only by economic variables but also by psychological, cognitive, and social factors. Prospect theory (Kahneman and Tversky, 1979) highlights individuals’ sensitivity to losses, while framing theory (Tversky and Kahneman, 1981) shows how decision-making is shaped by information presentation. Additional research has identified the role of cognitive biases, such as overconfidence, herding, and representativeness, in influencing investor behavior (Rabin, 1998; Rabin and Thaler, 2001). Empirical studies indicate that individuals often make decisions without full awareness of these biases, although increased awareness may lead to attempts to mitigate their effects (Phan and Zhou, 2014; Glaser and Weber, 2007). Research conducted in Turkey similarly shows that demographic factors influence risk perception and investment tendencies (Usul et al., 2002; Uluyol and Ayna, 2022). More recent studies on cryptocurrency markets highlight the role of psychological traits such as overconfidence, emotional satisfaction, environmental pressure, and self-control in shaping investment behavior (Sudzina et al., 2021; Pilatin and Dilek, 2024; Robba et al., 2023; Bland et al., 2024). Recent evidence further suggests that cryptocurrency investors exhibit distinctive demographic and emotional profiles, and that participation in cryptocurrency markets may also be associated with broader social and identity-related dynamics. For example, demographic characteristics and investor sentiment have been shown to influence patterns of engagement and behavioral tendencies in cryptocurrency markets (Campino and Yang, 2024), while cryptocurrency participation has also been linked to gendered forms of risk-taking, identity expression, and social status concerns, particularly among male investors (Cassino, 2023). Furthermore, research suggests that cryptocurrency investment may be associated with psychological risks such as stress and addiction (Jain et al., 2025). These findings collectively indicate that financial decision-making should be understood as a psychologically embedded process influenced by multiple interacting factors. However, existing studies have largely examined cryptocurrency investment through behavioral finance frameworks, while the psychological mechanisms underlying lived experience, identity construction, emotional adaptation, and behavioral transformation remain insufficiently explored.

Existing studies have substantially advanced understanding of cryptocurrency investment by examining behavioral biases, emotional reactions, social influence, and demographic characteristics. However, these dimensions have largely been investigated as separate explanatory mechanisms, providing only limited insight into how they interact and evolve throughout investors’ lived experiences under conditions of persistent uncertainty. Consequently, cryptocurrency investment remains insufficiently understood as an integrated socio-psychological adaptation process through which emotional regulation, coping strategies, social interaction, and identity construction develop together over time. Addressing this gap, the present study adopts a phenomenological approach to explore how these interconnected processes are experienced, interpreted, and negotiated by investors in their everyday lives, thereby offering a more integrated understanding of cryptocurrency investment as a lived experience rather than a set of isolated behavioral responses. Furthermore, by focusing on individuals living in rural areas of Türkiye, the study extends current knowledge by providing insights into an investor population that remains relatively underrepresented in the cryptocurrency literature. Taken together, these characteristics suggest that cryptocurrency investment cannot be adequately understood by examining emotional, cognitive, or social mechanisms in isolation. Instead, investors’ experiences emerge through the continuous interaction of these processes as they confront uncertainty, interpret market events, regulate emotions, seek social support, and gradually reconstruct their identities. This integrated perspective provides the conceptual foundation for the theoretical framework developed in the present study and guides the interpretation of the empirical findings presented in the following sections.

### Theoretical framework

Although behavioral finance provides valuable insights into investment decision-making, it offers only a partial explanation of how investors experience and adapt to persistent uncertainty in cryptocurrency markets. Understanding this process requires an integrated theoretical perspective that captures not only emotional responses but also the ways in which individuals regulate those emotions, develop coping strategies, interact with others, and gradually reconstruct their identities through investment experiences (Gross, 1998; Lazarus and Folkman, 1984; Tajfel and Turner, 1979). Accordingly, the present study combines Emotion Regulation Theory, Stress and Coping Theory, and Social Identity Theory into a single analytical framework. Rather than treating these theories as independent explanations, they are conceptualized as complementary and sequential perspectives that together explain how investors adapt psychologically and socially to uncertainty over time. This integrated framework also guides the interpretation of the empirical findings presented in the following sections. More specifically, Emotion Regulation Theory provides the analytical lens for understanding participants’ emotional responses to market volatility and their gradual development of emotional regulation strategies (Gross, 1998; Gross and John, 2003). Stress and Coping Theory explains how these emotional responses evolve into adaptive coping strategies that enable investors to manage persistent uncertainty and psychological stress (Lazarus and Folkman, 1984; Folkman and Moskowitz, 2004). Social Identity Theory, in turn, helps interpret how continued investment experiences and social interactions become incorporated into participants’ identities and influence their sense of belonging within cryptocurrency communities (Tajfel and Turner, 1979; Haslam, 2004). Finally, community influence provides the broader socio-psychological context within which emotional regulation, coping, and identity construction are continuously shaped through interactions with peers, online communities, and digital financial environments (Papadamou et al., 2023; Aebli et al., 2025).

### Emotion Regulation Theory

Emotion regulation constitutes one of the central psychological processes through which individuals manage emotional experiences in uncertain and stressful environments. According to Emotion Regulation Theory, individuals do not passively experience emotions; rather, they actively monitor, modify, and regulate emotional responses in accordance with situational demands and personal goals (Gross, 1998). This perspective is particularly relevant in cryptocurrency markets, where investors are frequently exposed to rapid price fluctuations, uncertainty, and emotionally charged information flows. Under such conditions, emotional reactions such as fear, anxiety, excitement, regret, and frustration may substantially influence decision-making processes.

From this perspective, investment behavior can be understood not only as a response to objective market conditions but also as a consequence of how investors regulate emotional experiences arising from those conditions. Individuals who successfully regulate emotional responses may be more capable of maintaining consistency in their investment strategies, whereas difficulties in emotional regulation may contribute to impulsive decisions, panic selling, excessive risk-taking, and emotionally driven trading behavior. Previous research has demonstrated that individual differences in emotion regulation influence decision quality, behavioral self-control, and adaptation to stressful circumstances (Gross and John, 2003; Gross, 2015). Consequently, emotion regulation provides an important theoretical lens for understanding how investors adapt to uncertainty and maintain psychological equilibrium in highly volatile financial environments (Gross, 1998; Gross and John, 2003). This perspective is particularly relevant for understanding emotionally driven investment behaviors such as panic selling, impulsive trading, and excessive risk-taking frequently reported in cryptocurrency markets. However, regulating emotional responses alone does not fully explain how investors remain engaged in highly uncertain cryptocurrency markets. Emotional regulation represents one component of a broader adaptation process in which individuals also evaluate stressful situations, develop coping strategies, and gradually adjust their behavior (Gross, 1998; Gross and John, 2003). Understanding this progression therefore requires extending the analysis beyond emotional regulation to include the coping processes through which investors respond to persistent uncertainty (Lazarus and Folkman, 1984; Folkman and Moskowitz, 2004).

### Stress and Coping Theory

While emotion regulation focuses on the management of emotional experiences, Stress and Coping Theory explains how individuals respond to stressful situations more broadly. Lazarus and Folkman (1984) conceptualized coping as the cognitive and behavioral efforts individuals employ to manage demands that are perceived as exceeding available resources. According to this framework, individuals typically rely on two broad categories of coping strategies: problem-focused coping, aimed at addressing the source of stress, and emotion-focused coping, aimed at managing the emotional consequences of stressful experiences. The theory further suggests that individuals continuously evaluate environmental demands and their available resources through cognitive appraisal processes, which subsequently shape coping responses (Lazarus and Folkman, 1984).

The cryptocurrency investment context represents a setting in which both forms of coping may emerge simultaneously. Investors may engage in problem-focused coping by seeking information, developing investment rules, diversifying portfolios, or implementing risk-management strategies. At the same time, they may rely on emotion-focused coping through reassurance-seeking, emotional distancing, acceptance, or cognitive reframing of losses. Because cryptocurrency markets involve persistent uncertainty, fluctuating risk perceptions, and recurring exposure to financial gains and losses, coping processes become central to understanding how investors maintain engagement, recover from adverse experiences, and adapt to market volatility over time. Previous studies indicate that effective coping strategies enhance resilience, facilitate adaptation to uncertainty, and reduce the psychological consequences of stressful events (Folkman and Moskowitz, 2004; Carver and Connor-Smith, 2010). Therefore, coping theory offers a valuable framework for interpreting how investors respond to uncertainty and psychological stress within highly volatile financial environments. Although coping strategies help individuals manage uncertainty and maintain psychological resilience (Lazarus and Folkman, 1984; Folkman and Moskowitz, 2004), adaptation is not solely an individual process. As investors repeatedly interact with other market participants, online communities, and broader investment cultures, these experiences gradually influence how they perceive themselves and their role within the cryptocurrency ecosystem. Consequently, understanding long-term adaptation also requires examining the identity-related processes through which investment experiences become incorporated into individuals’ self-concepts (Tajfel and Turner, 1979; Haslam, 2004).

### Social Identity Theory

Investment behavior is not solely shaped by cognitive and emotional processes but is also embedded within broader social and identity-related dynamics. Social Identity Theory proposes that individuals derive part of their self-concept from membership in social groups and from the meanings associated with those group affiliations (Tajfel and Turner, 1979). Through this process, individuals develop social identities that influence perceptions, attitudes, behaviors, and patterns of interaction. Group-based identification may provide individuals with a sense of belonging, social meaning, and self-definition, which can subsequently influence decision-making and behavioral tendencies (Haslam, 2004).

In the context of cryptocurrency investment, participation may gradually evolve beyond a purely economic activity and become integrated into an individual’s sense of self. As investors accumulate experience, interact with other market participants, and develop investment-related beliefs and practices, they may increasingly internalize an investor identity. This identity can influence how market events are interpreted, how risks are perceived, and how individuals respond to success, failure, and uncertainty. Prior research suggests that identity-related processes play an important role in shaping attitudes, behavioral commitment, and long-term decision-making patterns (Haslam, 2004; Stets and Burke, 2000). Social Identity Theory therefore provides a useful framework for understanding the identity-related transformations, internal conflicts, and evolving self-perceptions reflected in investors’ lived experiences. Taken together, these theoretical perspectives suggest that cryptocurrency investment should not be understood as a series of isolated emotional, cognitive, or social events. Rather, investors’ lived experiences reflect a continuous adaptation process in which emotional regulation, coping strategies, and identity construction mutually reinforce one another over time (Gross, 1998; Lazarus and Folkman, 1984; Tajfel and Turner, 1979). Because these processes unfold within digitally connected investment communities, it is also necessary to consider the broader social environments through which information, trust, and collective meanings are constructed (Papadamou et al., 2023; Aebli et al., 2025). This provides the rationale for considering community influence as an integral component of the present theoretical framework.

### Community influence and collective behavior in cryptocurrency markets

Building upon the integrated theoretical framework outlined above, community influence represents the broader social context within which emotional regulation, coping processes, and identity construction unfold. Unlike traditional investment environments, cryptocurrency ecosystems are characterized by continuous interaction through online platforms such as Reddit, Telegram, Discord, and X. These communities facilitate information exchange, collective interpretation of market events, and the formation of shared narratives among investors (Papadamou et al., 2023; Garcia and Schweitzer, 2015). Within these environments, investors may develop strong psychological attachments to specific cryptocurrency projects and online communities. Drawing on social identity perspectives, recent research has shown that meaningful social interactions, perceived social rewards, positive future expectations, and investment involvement contribute to the development of identity fusion within cryptocurrency communities (Aebli et al., 2025). Identity fusion refers to a strong alignment between personal identity and group identity, resulting in deep emotional commitment and a heightened sense of belonging to a particular community. In highly engaged cryptocurrency communities, investors may therefore maintain loyalty and commitment even during periods of substantial uncertainty and financial loss (Aebli et al., 2025).

In addition to community-based influences, social media personalities known as “finfluencers” have become increasingly important actors in shaping investors’ perceptions and financial decision-making processes. Finfluencers disseminate investment-related information through digital platforms and often influence how individuals interpret financial opportunities, risks, and market developments. Hayes and Ben-Shmuel (2024) argue that finfluencers do not merely communicate financial information but actively shape financial subjectivities through digital storytelling, participatory engagement, and the construction of relatable financial identities. Consequently, investment behavior in cryptocurrency markets may be influenced not only by market information but also by community participation, identity processes, and the social influence exerted by digital financial opinion leaders.

Taken together, cryptocurrency communities, identity fusion processes, and finfluencer influence highlight the importance of examining cryptocurrency investment as a socially embedded phenomenon. These perspectives complement Emotion Regulation Theory, Stress and Coping Theory, and Social Identity Theory by providing a more comprehensive understanding of how social interaction, community attachment, identity formation, and digital influence shape investors’ lived experiences. Accordingly, cryptocurrency investment can be understood not only as a financial activity but also as a social and psychological process in which collective identities, emotional bonds, and digitally mediated interactions play a significant role in shaping behavior (Aebli et al., 2025; Hayes and Ben-Shmuel, 2024; Papadamou et al., 2023).

### Methods

This research was conducted using a phenomenological approach. Participants consisted of 15 individuals who had actively invested in cryptocurrency within the last year. Although data saturation was reached after the fifteenth interview, four additional interviews were conducted to confirm thematic stability and strengthen the robustness of the findings. The study was completed with a total of 19 participants. In qualitative phenomenological studies, sample size typically ranges from 5 to 25 individuals (Creswell, 2013; Guest et al., 2006). Participants were selected using purposive sampling. The inclusion criteria for sampling were having at least 12 months of investment experience in the cryptocurrency market, voluntarily participating in the study, and being able to communicate effectively in Turkish. Individuals who did not invest in cryptocurrency and those who did not possess sufficient expressive skills were not included in the study. In qualitative research, purposive sampling contributes to obtaining in-depth data appropriate to the research objective by ensuring that individuals with specific characteristics participate in the study (Patton, 2002; Creswell, 2013; Uzun et al., 2025). Furthermore, establishing clear inclusion and exclusion criteria enhances the transparency and reliability of the data collection process (Guest et al., 2006). In addition to purposive sampling, a referral-based recruitment strategy was employed. After the initial participants were identified, some participants recommended other cryptocurrency investors who met the inclusion criteria. This approach facilitated access to individuals with relevant experience and helped ensure the richness of the data collected.

The study was conducted in 2025 with individuals living in the Eastern Black Sea Region of Turkey. In selecting participants, care was taken to include individuals from different ages, occupational, and gender groups to reflect psychological and behavioral diversity. Descriptive data regarding the sample are presented in Table 1. The semi-structured interview technique, one of the qualitative data collection methods, was used in the research. During the process of creating the interview form, the opinions of two academics specializing in finance and psychology were sought, and the content and wording of the form were updated based on the feedback received. The data collection process was conducted by a researcher with doctoral-level expertise in finance. The researcher was male and conducted all interviews individually. No individual contact was made with the participants prior to the interviews. Although most participants had general knowledge about the researcher’s academic background, this did not affect the data collection process. Before the interviews, the purpose and scope of the study were clearly explained to the participants, and information was provided about the researcher’s role and approach. All interviews were conducted in Turkish, which was the native language of all participants. Audio recordings were transcribed verbatim in Turkish immediately after data collection. For publication purposes, participant quotations presented in the manuscript were translated into English by the researchers. Particular attention was paid to preserving the original meaning, context, and emotional content of participants’ statements during the translation process. Minor linguistic adjustments were made solely to improve readability without altering the substantive meaning of the narratives. To improve readability in the English-language manuscript, minor linguistic adjustments were made to selected quotations during the translation process. These edits were limited to grammatical clarification and language fluency and did not alter the original meaning or substance of participants’ statements.

**TABLE 1:** Participant characteristics.

Participant number	Age	Gender	Marital status	Educational status	Investment period (years)
K1	55	Male	Married	Doctorate	2 years
K2	39	Male	Married	Master’s degree	5 years
K3	40	Male	Married	Bachelor’s degree	5 years
K4	41	Male	Married	University	6 years
K5	40	Male	Single	Master’s degree	5 years
K6	38	Male	Single	Secondary school	4 years
K7	33	Female	Married	Bachelor’s degree	4 years
K8	38	Male	Married	Master’s degree	5 years
K9	42	Male	Married	Secondary school	7 years
K10	40	Male	Married	Master’s degree	3 years
K11	31	Male	Single	Secondary school	2 years
K12	35	Male	Married	Bachelor’s degree	6 years
K13	42	Female	Married	Secondary school	7 years
K14	38	Male	Married	Bachelor’s degree	5 years
K15	30	Male	Single	Bachelor’s degree	4 years
K16	28	Male	Single	Bachelor’s degree	2 years
K17	36	Male	Married	Master’s degree	3 years
K18	34	Male	Married	Bachelor’s degree	5 years
K19	37	Male	Married	Bachelor’s degree	6 years

## Phenomenological method

The study design, data collection process, and analysis procedures were conducted and reported in accordance with the Consolidated Criteria for Reporting Qualitative Research (COREQ) guidelines (Tong et al., 2007). The completed COREQ checklist is provided in Appendix 1.

### Interview guide development

The interview guide was developed based on methodological recommendations for semi-structured interviews (Patton, 2002; Creswell, 2013; Turner, 2010; Kallio et al., 2016) and relevant theoretical perspectives from the behavioral finance and cryptocurrency literature. Specifically, the guide was informed by Prospect Theory (Kahneman and Tversky, 1979), Herd Behavior Theory (Bikhchandani et al., 1992; Hirshleifer and Teoh, 2003), the Adaptive Markets Hypothesis (Lo, 2017), Behavioral Finance 2.0 (Statman, 2019), Emotion Regulation Theory (Gross, 1998), Stress and Coping Theory (Lazarus and Folkman, 1984), and Social Identity Theory (Tajfel and Turner, 1979). In this context, answers were sought to the following questions.

– What emotions did you experience when you first started investing, and how did these emotions change over time? Can you provide an example to illustrate this change?
– Who in your circle influenced your decision to invest in cryptocurrency? How did comments on social media or from your close circle guide you?
– What emotions did you experience in the face of sudden market fluctuations? How did these emotions affect your decision-making process?
– How did you begin making investment decisions during this process? Did you initially proceed intuitively, or did you become more planned and analytical over time?
– What did the cryptocurrency investment process teach you? What was the most significant change for you as a result of this experience?

## Data analysis

The analysis process for this study was structured based on Colaizzi (1978) phenomenological approach and followed a seven-stage systematic analysis method (Morrow et al., 2015). The interview transcripts were analyzed repeatedly by the primary researcher in order to gain a deep understanding of the participants’ lived experiences. First, significant statements were identified and meaningful units were extracted while preserving their original context. These meaning units were then organized into codes, which were subsequently grouped into sub-themes and broader themes based on conceptual similarities and shared meanings.

To enhance the credibility and trustworthiness of the findings, the resulting codes, sub-themes, and themes were reviewed by a second academic with expertise in qualitative research and behavioral sciences. Any differences in interpretation were discussed until consensus was reached. The themes were derived inductively from participants’ narratives rather than being predetermined. Although the interview guide was informed by relevant theoretical perspectives, these theories were used only to support question development and did not determine the coding structure or thematic framework. Themes, sub-themes, and codes were generated inductively from participants’ accounts during the analysis process. Throughout the analysis process, each theme was supported by direct participant quotations to ensure transparency and allow readers to understand how the findings emerged from the data (Yıldırım et al., 2021). Because the study adopted a phenomenological approach, the purpose of involving a second academic was not to calculate a statistical intercoder reliability coefficient but to enhance interpretive credibility. The second reviewer independently examined the coding structure, theme definitions, and representative quotations. Any discrepancies in coding or thematic interpretation were discussed collaboratively until consensus was achieved. This consensus-based review process is consistent with recommendations for qualitative phenomenological research that prioritize interpretive agreement and reflexive dialogue over quantitative reliability indices (Lincoln and Guba, 1985; Creswell, 2013). Several strategies were employed to enhance the trustworthiness of the findings. An audit trail documenting coding decisions, theme development, and analytical reflections was maintained throughout the study. In addition, emerging codes, sub-themes, and themes were periodically reviewed with an independent academic expert experienced in qualitative research and behavioral sciences. These procedures contributed to the transparency, consistency, and credibility of the analytical process and helped minimize potential researcher bias (Lincoln and Guba, 1985).

## Ethical aspects of the research

The study was approved by the Ethics Committee of Gümüşhane University (Approval No: E-95674917-108.99-333503). Prior to data collection, all participants were informed about the purpose and procedures of the study, and written informed consent was obtained. Participation was entirely voluntary, and participants were informed that they could withdraw from the study at any stage without providing a reason. No financial or material incentives were offered in exchange for participation. All interviews were conducted confidentially, and participants’ personal information was anonymized during transcription and reporting. Data were stored in secure and encrypted digital environments accessible only to the research team. Ethical principles were adhered to throughout the study, and all procedures were conducted in accordance with the Declaration of Helsinki and relevant national ethical guidelines.

### Findings

According to the descriptive findings, the average age of the participants was 37 (SD ≈ 6.6), with individuals in their thirties and forties constituting the majority of the group. This indicates that the participants were in the middle of their careers. The average investment period in the cryptocurrency market was determined to be 4.5 years (SD ≈ 1.5). This result reveals that participants were exposed to market fluctuations to a certain extent and gained experience. The detailed demographic characteristics of the participants are presented in Table 1.

Qualitative data obtained from semi-structured interviews in the study were examined to reveal meaningful patterns; accordingly themes, sub-themes and related codes were systematically structured (Table 2).

**TABLE 2:** Reflections of psychological and behavioral experiences of individuals investing in cryptocurrency in an uncertain environment.

Themes	Sub-themes	Codes
1. Emotional and cognitive processes changing over time	A. From inception to maturity B. Personal transformation	A 1. Excitement and curiosity A 2. Anxiety arising from uncertainty A 3. Intuitive decision-making A 4. Searching for a new opportunity A 5. Transition to analytical thinking A 6. Better ability to weigh risks B 1. Confidence gained through experience B 2. Learning from losses B 3. Comparing the past self with the present self B 4. Increased emotional awareness B 5. Evolution of investment strategy B 6. Developing a long-term perspective
2. The impact of social interactions on investment motivation	A. Environmental influences B. Trust dynamics	A 1. Encouragement from family and friends A 2. Investment recommendations on social media A 3. Falling prey to herd behavior A 4. Tendency to delegate decision-making power to others A 5. Explicit encouragement A 6. Implicit guidance A 7. Decisions being shaped by external influences B 1. Support from the environment for the investor identity B 2. Judgmental attitudes of the environment B 3. Building trust B 4. Loss of trust B 5. Conscious or unconscious influences B 6. The effect of environmental pressure on decision-making
3. Internal emotional responses to uncertainty	A. Risk and decision dilemmas B. Emotional outcomes	A 1. Sudden changes in risk perception A 2. Being caught between fear and inaction A 3. Panic selling A 4. Irrational decisions A 5. The dilemma of whether it is the right time A 6. Making quick decisions under uncertainty A 7. Indecision A 8. Fear of making mistakes B 1. Restlessness B 2. Feeling of inadequacy B 3. Regret B 4. Post-traumatic evaluation
4. Coping with isolation and the search for support	A. Presence of support B. Lack of support	A 1. Increased confidence A 2. Motivation A 3. Understanding environment A 4. Empathetic communication A 5. Guidance from a trusted person B 1. Feeling of loneliness B 2. Inability to share B 3. Carrying the emotional burden alone B 4. Family pressure B 5. Psychological relief B 6. Contributing to stress management
5. Identity dilemma and transformation in self-awareness	A. Investor identity and internal conflicts B. Life priorities	A 1. Identification with investor identity A 2. Self-confidence through financial success A 3. Need for control A 4. Feeling of burnout A 5. Conflict between emotional identity and analytical identity A 6. Questioning: “I became an investor, but have I lost other aspects of myself?” A 7. Identity restructuring A 8. Personal empowerment B 1. Life priorities changed by investment B 2. Changes in behavioral patterns B 3. Increased self-awareness B 4. Long-term identity transformation

Table 2 systematically presents the themes, sub-themes, and codes derived from the participants’ experiences. However, these findings not only provide a classified overview but also illustrate the interrelationships and holistic patterns emerging across the themes. To visually integrate these relationships, a conceptual model was developed. As shown in Figure 1, the emotional and cognitive processes initially experienced by investors were shaped over time through social interactions, internal responses to uncertainty, and social support systems, ultimately contributing to transformations in identity perception and life priorities. Thus, the multidimensional structure of the qualitative findings is presented as an integrated conceptual framework rather than merely a descriptive categorization (Creswell, 2013; Moustakas, 1994).

**FIGURE 1:**
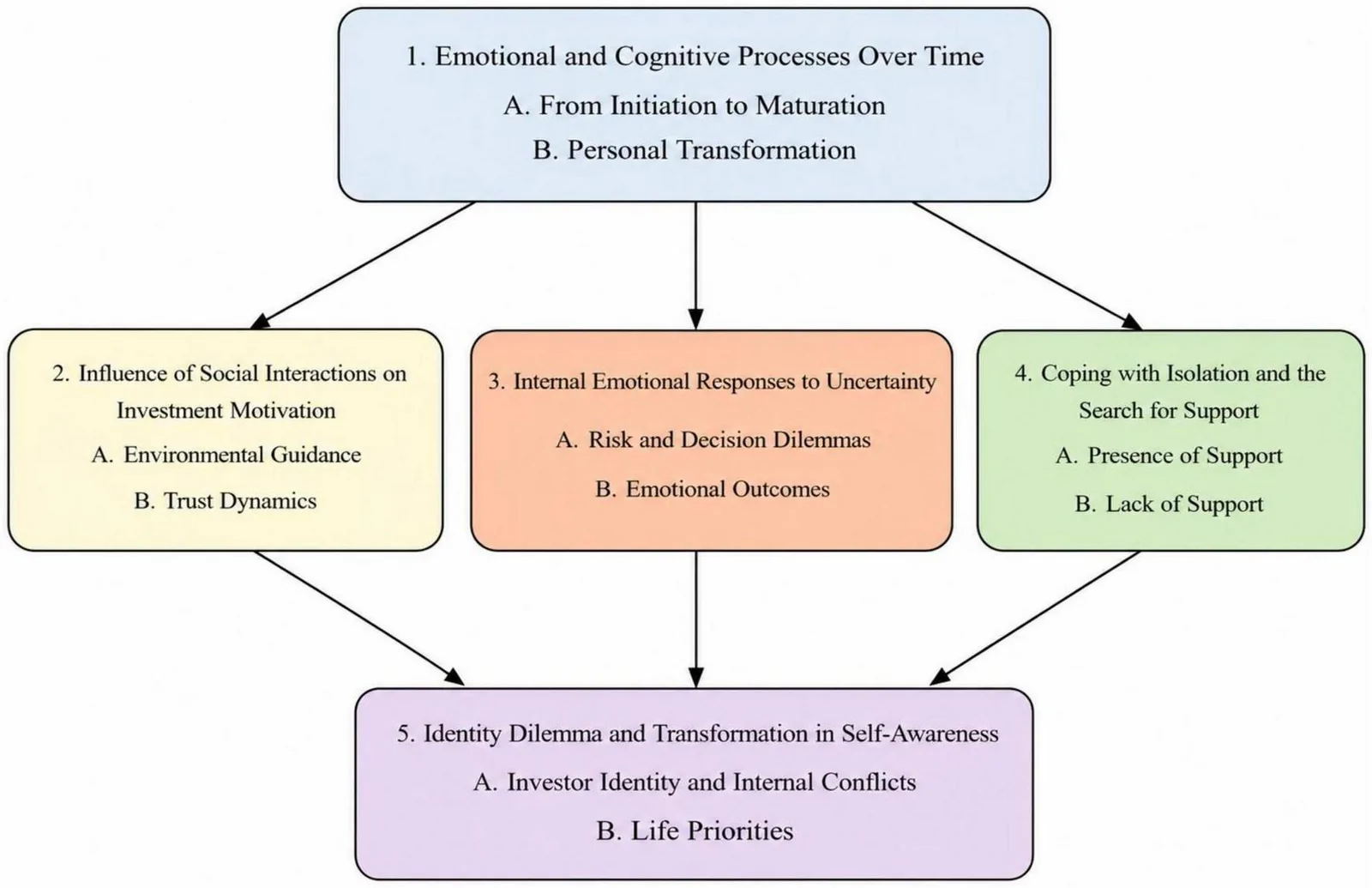
Conceptual model of the relationships among themes emerging from cryptocurrency investors’ lived experiences.

The alphanumeric labels presented after each quotation indicate the participant identifier and the corresponding analytical codes generated during the coding process. For example, “K8: 2A1” refers to Participant 8 and a specific code associated with Theme 2, Sub-theme A. These codes are provided to enhance analytical transparency and to demonstrate the connection between participants’ narratives and the thematic structure developed during data analysis.

Figure 1 presents the conceptual relationships among the themes derived from the phenomenological analysis. The arrows indicate the directional flow of participants’ experiences as interpreted from the data. The model suggests that emotional and cognitive processes developing over time influence how individuals respond to social interactions, uncertainty, and social support mechanisms. These experiences, in turn, contribute to the formation of investor identity, self-awareness, and changes in life priorities. The arrows do not imply strict causal relationships; rather, they represent conceptual and experiential connections identified across participants’ narratives. Thus, the model should be interpreted as a thematic representation of the dynamic and interconnected nature of cryptocurrency investment experiences.

### Theme 1. Emotional and cognitive processes changing over time sub-theme 1. From inception to maturity

Cryptocurrency investment clearly reveals the emotional and cognitive transformation individuals undergo over time. Initially, most investors act with intense excitement and curiosity, emboldened by the prospect of quick profits offered by the market. However, high volatility and uncertainty add anxiety and concern to these positive emotions. Research shows that cryptocurrency investors, particularly in the early stages, act on intuitive and emotional reflexes, which can result in panic selling and irrational decisions (Delfabbro et al., 2021b). Over time, experience reveals that decisions based solely on emotions are unsustainable, prompting investors to develop a more rational approach. Indeed, behavioral finance literature emphasizes that investors learn from their losses, reshape their risk perceptions, and turn to strategic thinking (Kahneman and Tversky, 1979; Statman, 2019). During this transformation process, investors, who initially tend to evaluate opportunities hastily, gradually begin to better understand market dynamics and adopt analytical thinking. Recent review studies reveal that individuals participating in crypto markets have developed their ability to manage emotional fluctuations and gained cognitive resilience against volatility (Jain et al., 2025). Cryptocurrency investment is not only an economic process but also one with psychological and behavioral dimensions. This process has become a multi-layered learning space that strengthens individuals’ capacity to cope with uncertainty, balance their emotional responses, and develop long-term strategies (Delfabbro et al., 2021b; Jain et al., 2025). These findings are consistent with Emotion Regulation Theory, which suggests that individuals gradually learn to regulate emotional responses in uncertain and stressful environments (Gross, 1998). The transition from intuitive reactions to analytical thinking observed among participants reflects increasing emotional regulation and behavioral adaptation. These preliminary findings suggest that participants’ early investment experiences were characterized by a gradual shift from emotionally driven and intuitive decisions toward more reflective and analytical approaches. Rather than representing an isolated cognitive change, this transformation appears to provide the foundation upon which subsequent experiences of social influence, coping, and identity development are built (Gross, 1998; Lazarus and Folkman, 1984). The following participant narratives illustrate how this emotional and cognitive transition unfolded through lived experience.

*“When I started investing in cryptocurrency about two years ago, my emotions were like a storm: excitement, curiosity, and a bit of fear. The idea of making huge profits from something new like Bitcoin or Ethereum was incredibly appealing. I remember staying up late into the night watching YouTube videos and browsing Reddit forums; it felt like I was on the brink of a financial revolution. However, even investing the £10 I had saved felt like gambling. Every price increase filled me with excitement, while every drop created a knot in my stomach. Over time, these intense emotions transformed into cautious optimism. The ups and downs of the crypto markets taught me resilience. For instance, at the start of 2023, swept up in the hype of a Telegram group, I invested in a promising altcoin. Within a week, surged by 50%, and I felt invincible, even fantasizing about quitting my job. But then, following a hack rumor, the coin plummeted by 70% overnight. That loss stung, but it was a turning point. I realized that emotional highs made me overconfident, while lows pushed me to research more carefully. Now, I approach my investments with discipline; I clarify my entry and exit points. The excitement is still there, but it’s balanced by my respect for the market’s volatility. That altcoin crash wasn’t just a financial lesson; it was also a psychological lesson in trusting data over emotions.” (K1: 1A1, 1A2, 1A3, 1A4, 1A5)*

*“During my initial investment period, excitement and uncertainty were intertwined. The fear of losing was as intense as the hope of winning. Over time, as I gained experience, I developed a more cautious and patient approach. For example, in my first investment, I expected high short-term gains and acted hastily; when I lost money, I learned that I shouldn’t act without understanding the dynamics of the market. Now I make my decisions based more on analysis and long-term thinking.” (K5: 1A1, 1A2, 1A3, 1A5, 1A6)*

*“When I first entered crypto, I was quite excited, and frankly, a bit nervous. Everyone seemed to be making a lot of money, but at the same time, there were so many unknowns. In the beginning, I dealt with short-term buying conditions, but constantly monitoring the market and making quick decisions was exhausting. Then I realized that patience pays off in this business. For example, during a major downturn, while everyone else was panicking and selling, I held onto what I had, but I didn’t reap the rewards of that patience like others did. This has sometimes led me to struggle with controlling my emotions.” (K6: 1A1, 1A2, 1A3, 1A6)*

### Sub-theme 2. Personal transformation

The experiences gained reveal that the investment process in cryptocurrency markets involves not only financial but also psychological dimensions. Strategies developed by investors over time, such as stop-loss orders, target price setting, and disciplined trading rules, limit the impact of emotional fluctuations on decision-making processes. Indeed, research shows that investors’ increased ability to manage extreme emotions such as fear and excitement contributes to reshaping their perception of risk and enabling them to make more cautious, long-term decisions (Delfabbro et al., 2021b). It is noted that individuals gain emotional awareness after experiencing losses, and this awareness strengthens strategic thinking and a long-term perspective (Barberis and Thaler, 2003; Statman, 2019). Furthermore, a recent review study has revealed that individuals participating in cryptocurrency markets gain psychological resilience and become more conscious in regulating their emotional responses (Jain et al., 2025). Thus, the investment process is not limited to economic gains or losses; it also becomes an area of personal transformation that allows individuals to develop cognitive flexibility, balance their emotions, and strengthen their rational decision-making skills (Delfabbro et al., 2021b; Jain et al., 2025). The observed shift from emotionally driven decisions to disciplined and strategic investment behavior is also consistent with Stress and Coping Theory. Participants increasingly adopted problem-focused coping strategies by learning from losses, developing structured decision rules, and strengthening emotional resilience (Lazarus and Folkman, 1984; Folkman and Moskowitz, 2004).

*“At first, my investment decisions were entirely intuitive. When I started in 2022, I didn’t even know what a whitepaper was. I bought coins based on my instincts, often getting caught up in hype. For example, I invested $200 in a coin because the Telegram group was very active and the name sounded promising. No research, just gut feeling. If the price rose, I jumped in, lured by the allure of quick profits. Losses piled up quickly; the coin lost 80% of its value in a month. Over time, I realized intuition wasn’t enough. After losing $300 on another hyped coin, I paused and reflected. I started watching YouTube videos on technical analysis; I learned terms like “support levels” and “RSI.” I tried to understand Ethereum’s technology by reading its roadmap, which gave me confidence to hold it long-term. By mid-2023, I had a system: I checked a coin’s market cap, team, and use case. For example, I bought Solana after comparing its transaction speeds to Ethereum’s and trusting its scalability. Now my approach is analytical but not rigid. I use data tools like CoinGecko and charts on TradingView, setting price alerts to avoid emotional buying. I allocate 70% of my portfolio to “safe” coins like Bitcoin and Ethereum, 20% to mid-sized projects, and 10% to high-risk bets. This plan was shaped by trial and error; early losses taught me diversification, while Ethereum’s steady gains taught me patience. I still feel intuitive urges, especially during market excitement, but I balance them with research. Last month, I almost jumped into a trending memecoin, but I backed out when I saw it had no real use case. Shifting from chasing feelings to building a strategy has boosted my confidence, but I’m still learning.” (K1: 1B1, 1B2, 1B3, 1B5)*

*“Of course, I have experienced losses from time to time; this led me to consider completely stepping away from the market for a short period. However, I soon realized this wasn’t a permanent solution. The fluctuations I experienced showed me that the real challenge in this field is not just financial, but also maintaining emotional balance. The disappointment and regret that follow losses, in particular, can create hesitation in the decision-making process. However, over time, I learned that controlling the intensity of emotions and taking more cautious steps is healthier in the long run. Therefore, when making decisions now, I try to consider not only market data but also my own emotional responses, aiming to adopt a more patient and disciplined approach during sudden fluctuations.” (K4: 1B1, 1B2, 1B3, 1B6) “In the early days, I made my investment decisions largely intuitively; I would quickly take positions based mostly on advice I heard from those around me or short-term expectations of growth. However, I saw more clearly, especially after experiencing some losses, that this approach was not sustainable. The experience I gained over time led me to adopt a more planned and analytical perspective. Now, when making decisions, I do not get carried away by momentary excitement; instead, I utilize technical analysis methods, current market news, and clues provided by historical data. Thus, I strive to make decisions based on more conscious and long-term strategies rather than intuitive actions.” (K5: 1B1, 1B2, 1B3, 1B4, 1B5)*

Taken together, Theme 1 demonstrates that participants’ cryptocurrency investment journeys evolved from emotionally driven and intuitive reactions toward increasingly reflective, disciplined, and strategic forms of decision-making. Importantly, this transformation did not occur in isolation. As participants gained experience, interactions with family members, peers, and digital investment communities increasingly influenced how they interpreted market information and evaluated investment opportunities. This progression provides the experiential foundation for the second theme, which examines the role of social interactions in shaping investment motivation.

### Theme 2. The impact of social interactions on investment motivation sub-theme 1. Environmental influences

The process of turning to cryptocurrency investment is shaped by the influences individuals receive from both their social circles and digital platforms. Direct or indirect encouragement from family and friends plays an important role in the starting point of investment behavior. Success stories from people in one’s immediate circle strengthen curiosity and the perception of opportunity, paving the way for individuals to take the first step. Similarly, investment recommendations encountered on social media, posts by financial influencers, and content on platforms such as YouTube emerge as powerful guiding factors in investment decisions. The literature also emphasizes that guidance from social circles and digital media sources plays a decisive role in investor behavior, particularly increasing the tendency to succumb to herd mentality and delegate decision-making power to others (Hirshleifer and Teoh, 2003; Glaser et al., 2019). However, these initially effective influences are gradually being questioned by individuals, and investors are increasingly recognizing the importance of conducting their own analysis and making independent decisions. These findings support research suggesting that cryptocurrency investment decisions are embedded within digital communities where social interaction, information exchange, and collective sentiment influence behavioral tendencies (Papadamou et al., 2023; Garcia and Schweitzer, 2015). The influence of social media content creators and online communities further highlights the growing role of digitally mediated social influence in shaping investment behavior (Hayes and Ben-Shmuel, 2024). Indeed, research shows that as investors gain experience, they reassess the reliability of social influences and place greater weight on individual information processing (Baker and Ricciardi, 2014; Chen et al., 2022).

*“Success stories I encountered on social media initially influenced my decision to invest in cryptocurrency. In particular, posts by certain financial content creators on Twitter and stories shared by friends in my immediate circle who were investing increased my interest in this area and accelerated my decision-making process. However, over time, I realized that everyone’s risk perception, expectations, and strategies differ. Therefore, while others’ guidance was important at the beginning, I realized that it was not healthy to act without doing my own research. Now, while I take advice from social media or my circle into consideration, I take care to base my decisions more on my own analysis.” (K8: 2A1, 2A2, 2A3, 2A5, 2A6, 2A7)*

*“My family was quite cautious and skeptical about this; therefore, their influence on my decision-making process was limited. However, there were a few people in my circle of friends who were investing, and sharing their experiences piqued my curiosity. However, what influenced me most were the YouTube videos of a female investor I came across on social media. I found her narrative style reliable and explanatory; her ability to convey how the market works in simple terms was decisive in my decision. Over time, I realized that although such sources are helpful, they are not sufficient on their own; despite creating a sense of trust, I learned that I needed to base my final decision on my own research and assessments.” (K9: 2A1, 2A2, 2A4, 2A5, 2A6)*

*“Initially, I saw many posts about cryptocurrencies on social media, but I didn’t take them too seriously. My interest really grew when a friend mentioned the subject. For example, when I saw that investing in a coin yielded higher returns than gold within two years, my curiosity was piqued. As I saw similar examples on social media and in various videos, the thought that ‘if I invest a small amount, I won’t lose much’ began to prevail. My friend and I decided to invest at the same time. Still, I didn’t approach the process too ambitiously; I acted more cautiously, considering the possible risks, and preferred to invest small amounts at first.” (K11: 2A1, 2A2, 2A3, 2A5, 2A6)*

### Sub-theme 2. Trust dynamics

The influence of social circles and media discourse is distinctly felt in the process of turning to cryptocurrency investment. The support of the environment for the investor identity, the sharing of success stories, and the promise of high returns inspires a sense of trust and curiosity in individuals. Indeed, the literature also indicates that the social environment functions as a trust-building element in investment decisions and shapes individuals’ perceptions (Shiller, 2015). Information and emotional content disseminated on social media platforms can facilitate the transfer of trust between individuals and strengthen investment intentions (Warkulat and Pelster, 2024; Guégan and Renault, 2021). However, judgemental attitudes or exaggerated rhetoric from the environment can also lead to a breakdown in this trust and push investors into a more cautious position. Investment decisions, which are initially guided by conscious or unconscious influences, undergo a transformation as the reality of external pressures is recognized over time. Research shows that statements such as “early birds get the worm” or “get rich quick” trigger herd behavior, but confronting market risks leads investors to make more independent and rational decisions (Hirshleifer and Teoh, 2003; Hoffmann et al., 2015). In this context, the cryptocurrency experience is considered not only a financial process but also a multidimensional learning space shaped by trust, social interactions, and cognitive awareness. From a theoretical perspective, these findings can be interpreted through Social Identity Theory, which suggests that individuals’ beliefs and behaviors are shaped by the social groups with which they identify (Tajfel and Turner, 1979). Trust, belonging, and social validation appear to influence not only investment decisions but also the development of investor identity. Collectively, these findings indicate that participants’ investment decisions were shaped not only by their own evaluations but also by ongoing interactions with family members, peers, online communities, and digital financial content. At the same time, participants increasingly recognized the limitations of relying solely on external guidance, prompting a gradual shift toward more independent decision-making (Baker and Ricciardi, 2014; Chen et al., 2022). The following narratives illustrate how social influence functioned both as a source of motivation and as a catalyst for developing greater personal responsibility in investment decisions.

*“My decision to invest in cryptocurrency was most influenced by my circle of friends and posts I encountered on social media. In particular, the stories of people claiming to have made high profits and news of sudden rises aroused a strong sense of curiosity in me. The saying ‘early birds catch the worm’ from someone close to me was also an important factor that encouraged me to make a quick decision. However, over time, I experienced that such guidance did not always reflect reality and sometimes even led to emotional haste. This process demonstrated the power of social influences on investment decisions while also making me realize how important it is to trust my own analysis.” (K5: 2B1, 2B3, 2B5, 2B6) “Initially, the guidance of some friends in my circle increased my interest in cryptocurrencies. During the same period, I also encountered numerous posts, comments, and information on social media; while some of these seemed appealing, such as, I eventually realized they could be manipulative. Therefore, I began to act more cautiously, especially during periods of sharp market fluctuations. I made an effort not to be influenced by others’ comments and suggestions, focusing instead on my own research. This allowed me to move away from making quick decisions based on external influences and toward taking more conscious steps grounded in my own analysis. Although my social circle and social media were quite influential at first, I learned over time that it was healthier to trust my own decisions.” (K6: 2B1, 2B3, 2B4, 2B5, 2B6)*

*“The biggest factor in my entry into the cryptocurrency market was the encouragement of my close friends. The frequently repeated statements in my circle, such as “I made this much money” or “those who get in become millionaires in a short time,” inevitably caught my attention and aroused my curiosity. Such narratives made me feel that social interactions can be strongly influential and create an invisible pressure. Naturally, these statements instill a desire to act quickly and fuel the fear of missing out. However, as I delved deeper, I realized that most of what was being said was exaggerated or one-sided. When confronted with the real dynamics of the market, I realized that the idea of making big profits the easy way did not reflect reality. This process led me to act independently of external influences and base my decisions more on my own research.” (K7: 2B1, 2B3, 2B4, 2B5, 2B6)*

Overall, Theme 2 demonstrates that social interactions functioned as both an entry point into cryptocurrency investment and an important source of learning throughout participants’ investment journeys. As participants gradually became less dependent on external advice and increasingly relied on their own judgment, they were also required to confront the emotional challenges associated with making independent decisions under conditions of persistent uncertainty. This transition naturally leads to the third theme, which explores how participants experienced, interpreted, and managed their internal emotional responses to uncertainty.

### Theme 3. Internal emotional responses to uncertainty sub-theme 1. Risk and decision dilemmas

Sudden fluctuations in the cryptocurrency markets directly affect investors’ emotional responses and decision-making processes. In the early stages, intense price movements caused sudden changes in risk perception, leaving investors torn between fear and inaction. Indeed, studies show that volatility increases investors’ fear of loss, leading to hasty or irrational behavior (Kahneman and Tversky, 1979; Shiller, 2015). Panic selling, particularly during sharp declines, often leads to irrational decisions, diverting investors from their long-term goals. During this process, the dilemma of “Is this the right time?” and the necessity of making quick decisions under uncertainty lead investors into indecision and trigger the fear of making mistakes. The literature emphasizes that investors are caught between emotional reactions and rational strategies when making decisions under uncertainty (Lo, 2017; Statman, 2019). Perceived unfairness increases risk-taking tendencies (Sekścińska and Rudzinska-Wojciechowska, 2021). These findings are strongly aligned with Stress and Coping Theory. Participants frequently described uncertainty as a psychological stressor and reported adopting coping strategies such as information seeking, risk management, and disciplined decision-making to reduce emotional distress (Lazarus and Folkman, 1984). These findings suggest that uncertainty was experienced not simply as a market characteristic but as an ongoing psychological challenge requiring continuous emotional adjustment. As participants accumulated experience, they increasingly described moving from reactive emotional responses toward more deliberate strategies for managing uncertainty (Lazarus and Folkman, 1984; Folkman and Moskowitz, 2004). The following participant accounts illustrate how this transition was experienced in everyday investment practice.

*“The cryptocurrency investment process taught me that having a long-term perspective is much healthier than getting caught up in short-term trends. In particular, I experienced the importance of investing at the right time, at appropriate price levels (buying at the bottom), and avoiding panic selling in the face of sharp market fluctuations. This process demonstrated that not only patience but also a disciplined strategy is indispensable for success. I also had the opportunity to experience the logic behind how the market operates, how risks should be managed, and in which situations opportunities can be evaluated. Over time, I realized that while the desire to make large profits quickly may be appealing, it often leads to the rapid depletion of existing savings. Therefore, I learned that in the investment process, it is necessary to prioritize patience over ambition, research over rumors, and planned strategies over random experiments. The biggest change for me is that I now make investment decisions based on analytical thinking and conscious risk perception rather than emotional reflexes.” (K11: 3A1, 3A3, 3A4, 3A5, 3A8)*

*“Sudden fluctuations in the crypto market are like emotional earthquakes, in my opinion. In the early days, Bitcoin falling 20% in a single day sent me into a panic; I was glued to my phone, constantly checking my portfolio. I felt helpless, as if my savings were evaporating. On the other hand, Ethereum’s 30% surge after a major update in 2024 filled me with excitement; I sent screenshots of my profits to my friends. These extremes fear and greed defined my first year. These emotions greatly influenced my decisions. During a crash in 2023, I sold half my Bitcoin at a loss; I feared it would fall further. Days later, it rebounded, and I berated myself. This fear-driven decision cost me hundreds of dollars. Conversely, during a bull market, I bought into a hyped memecoin at its peak out of greed; it crashed when the hype faded. These mistakes stemmed from emotions clouding my logic. Over time, I learned to manage these emotions. Now, during dips, I take a deep breath and check the fundamentals: Does the project still have strong developers or adoption? This is what allowed me to hold on during last month’s 15% Ethereum drop; it recovered within weeks. I also set rules like not checking prices after 9 PM to prevent panic selling at night. These strategies reduce emotional noise and help me focus on long-term trends. Still, volatility challenges me. A recent sharp drop triggered old anxieties, but instead of selling, I trusted my research and made a small dip purchase. The shift from reactive moves to proactive decisions reflects increased emotional confidence. I still feel fear or excitement, but I rein them in and channel them toward calculated risks.” (K1: 3A1, 3A2, 3A3, 3A4, 3A5, 3A6, 3A8)*

*“I can say that I experienced intense stress and uncertainty in the face of sudden fluctuations in the markets. In particular, the sharp drop in prices in a short period of time triggered feelings of anxiety and panic in me. In such situations, there were times when I struggled to maintain my composure and made impulsive selling decisions based on emotional reactions. These types of transactions made in a state of panic overshadowed the rational thought process and caused me to stray from my long-term goals. However, my experiences over time have shown me the importance of emotional security. I developed systematic strategies to reduce the internal conflict I felt in the face of market uncertainties. For example, stop-loss orders that trigger at specific losses and pre-determined target price levels limited my tendency to make emotional decisions, enabling me to act more consciously and strategically. This allowed me to shift toward a more consistent and rational risk management approach rather than reacting impulsively to market fluctuations.” (K8: 3A1, 3A2, 3A3, 3A4, 3A6, 3A8)*

*“When I first started investing, I was completely intuitive. I made snap decisions based on price fluctuations and often acted on my emotions. However, quickly losing my gains helped me realize that this approach was unsustainable. When investing, I now research the projects behind the coins, follow the teams, technologies, current news, and global developments. I particularly took care to critically analyze and interpret news on social media. With this development, I learned to control my emotional reactions and be patient. For example, instead of investing large amounts at once, I started making gradual purchases and identifying different entry points. This strategy allowed me to act without panicking, especially when prices fluctuated, and to make long-term investments without having to constantly monitor the market. I didn’t always get perfect results, but over time I became a more methodical investor.” (K16: 3A4, 3A5, 3A6, 3A8)*

### Sub-theme 2. Emotional consequences

Sudden fluctuations in cryptocurrency markets create intense unease in investors’ emotional world and directly affect their decision-making processes. Particularly in the early stages of investment experience, rapid price declines trigger feelings of inadequacy; the sense of being caught unprepared by the market limits the capacity for rational thinking. Indeed, research shows that the volatility of cryptocurrency markets increases investor anxiety, leading to irrational reactions (Stix, 2021). Panic selling as a result of sudden losses often brings feelings of regret and causes individuals to re-evaluate their investment strategies (Shiller, 2015). However, these emotional fluctuations not only lead to losses but also pave the way for learning and the development of strategic awareness. The literature emphasizes that investors become better at managing emotional fluctuations over time and develop risk hedging strategies (Statman, 2019). Post-stress assessments contribute to individuals acting in a more disciplined manner; mechanisms such as stop-loss orders or target price setting limit irrational behavior and pave the way for more rational decisions (Liu et al., 2022). Thus, fluctuations, which are initially a source of intense stress, gradually transform into a learning process that increases emotional resilience and reshapes risk perception (Lo, 2017). Similarly, the development of emotional resilience and the increasing ability to manage fear, anxiety, and regret support the central assumptions of Emotion Regulation Theory (Gross, 1998; Gross and John, 2003). Participants’ experiences suggest that emotional regulation becomes an important determinant of long-term investment behavior.

*“I can say that I experienced intense stress, anxiety and, at times, regret in the face of sudden fluctuations in the markets. Especially in the early stages of my investment experience, a sharp drop in prices would create a strong sense of panic in me. This state of panic made it difficult for me to analyze the situation calmly and often led me to rush into selling. Therefore, emotional reactions significantly limited my capacity for rational decision-making. These experiences made me realize the critical role of emotional security in the investment process. As I came to accept that markets are inherently volatile, I observed that my internal conflicts diminished and I developed a more balanced approach. For example, whereas in the past I would sell quickly out of fear of loss, I have now learned to minimize the impact of impulsive decisions by setting specific rules, such as stop-loss orders and pre-determined target prices. However, uncertainties about timing, namely the question of whether I am buying or selling at the right moment, still occupy my mind. However, the lessons learned from previous experiences have made it easier for me to take more planned and strategic steps rather than irrational reactions. This process has not been limited to financial results; it has also contributed to redefining my perception of risk and strengthening my emotional resilience.” (K5: 3B1, 3B2, 3B3, 3B4)*

*“Sudden fluctuations in the markets have been quite a jarring experience for me, especially in the early stages of the investment process. Sharp losses in value occurring within a short period of time brought with them intense stress and uncertainty; this often led me to react at a panic level and make hasty selling decisions. I observed that such panic behavior was usually followed by increased feelings of regret and anxiety. Thus, I experienced how emotional reactions could overshadow my rational decision making processes. However, the experience I gained during this process helped me understand that fluctuations are a structural reality of the market. Over time, I learned to approach these ups and downs more prepared and to control feelings of panic and anxiety. I now take care not to immediately sell in response to sudden fluctuations, but rather to act based on pre-determined stop-loss levels and target prices. As a result, through my experiences, I have strengthened my ability to manage internal conflicts and adopted a more systematic, planned, and rational approach in my decision-making processes. This transformation has increased my emotional resilience in the face of market uncertainties, while also contributing significantly to my personal development in conscious risk management.” (K6: 3B1, 3B2, 3B3, 3B4)*

*“Sudden fluctuations in the markets, especially in the early stages of investing, caused me intense stress, anxiety, and feelings of uncertainty. Seeing prices double in a short period was initially quite exciting, but the persuasion from my surroundings that “it will rise even more” clouded my rational assessment capacity and prevented me from realizing my profits. During this process, I experienced an internal conflict between the desire for quick gains and the fear of losing what I had earned. In particular, panic-driven sales during sudden drops led to capital losses and intense regret afterwards. However, these negative experiences made me realize the decisive role of emotional security in investment decisions. Over time, I developed systematic strategies such as stop-loss orders and target price setting; this allowed me to control my emotional reactions and adopt a more rational, disciplined, and long-term approach. Thus, market volatility is no longer merely a source of stress but has become a learning process that reshapes my perception of risk and strengthens my identity as an investor.” (K7: 3B1, 3B3, 3B4)*

Collectively, Theme 3 indicates that participants gradually developed more effective strategies for regulating their emotional responses to uncertainty. However, their experiences also revealed that successful adaptation was not achieved solely through individual emotional regulation. Many participants emphasized the importance of sharing their concerns, seeking reassurance, and receiving support from trusted others during periods of market volatility. This progression provides the basis for the fourth theme, which explores the role of social support in coping with uncertainty and strengthening psychological resilience.

### Theme 4. Coping with isolation and the search for support sub-theme 1. Presence of support

Participants frequently emphasized the importance of supportive relationships during periods of market uncertainty and volatility. Emotional support from family members, friends, and trusted individuals contributed to increased confidence, motivation, and psychological comfort. The opportunity to share concerns and discuss investment experiences helped participants manage stress more effectively and reduced the emotional burden associated with financial uncertainty. In several cases, supportive interactions facilitated emotional regulation and encouraged more disciplined decision-making. These findings suggest that social support functions as an important coping resource by strengthening psychological resilience and helping investors adapt to challenging market conditions. This interpretation is consistent with previous research highlighting the protective role of social support during stressful experiences (Cohen and Wills, 1985; Thoits, 2011) and with Stress and Coping Theory, which emphasizes the value of social resources in managing uncertainty and emotional strain (Lazarus and Folkman, 1984). Taken together, these findings indicate that coping with uncertainty extended beyond individual psychological efforts and frequently involved the availability or absence of supportive social relationships. Participants’ experiences demonstrate that emotional resilience was strengthened not only through personal coping strategies but also through trusted interpersonal connections (Cohen and Wills, 1985; Thoits, 2011). The following narratives illustrate how the presence or absence of social support shaped participants’ investment experiences.

*“When I first started, I had no knowledge or experience, so I was buying and selling coins based on hearsay, social media, and peer influence. I was completely unaware, and since I ended up with significant losses, I started experiencing stress and had issues with my family. Later, I realized this wasn’t the right way to do it and educated myself on technical and fundamental topics. As a result, I traded calmly without panic, and as I started making profits, my confidence and motivation increased.” (K13: 4A1, 4A2)*

*“I made my investment decision based on the prediction that the significant declines in the stock markets could be an opportunity. Initially, I acted based on intuition, consultation, and the belief that a great opportunity had arisen. However, in the subsequent period, the daily fluctuations in the investment began to cause unease, sometimes unhappiness, and sometimes weariness. Nevertheless, I decided to maintain my motivation and wait in line with my set goal. And as a result, guided by my family and after a year of waiting, I sold my holdings, believing that I had achieved the goal I had in mind.” (K14: 4A1, 4A2, 4A5)*

### Sub-theme 2. Lack of support

The cryptocurrency investment process can often become a psychologically challenging experience due to individuals’ tendency to feel isolated and unable to share their feelings. In particular, increasing losses lead to individuals bearing the emotional burden alone, making it difficult for them to seek support from their families and even causing them to avoid potential family pressure. This situation is consistent with findings in the literature that insufficient social support during stressful periods negatively affects individuals’ psychological well-being (Cohen and Wills, 1985; Thoits, 2011). Decisions made in the early stages based on advice from friends or relatives have evolved into more planned and analytical strategies over time as individual experience has increased. Research shows that social influences are dominant in individuals’ investment behavior at the outset, but as experience increases, individual decision-making capacity strengthens (Bikhchandani and Sharma, 2000). The flow of information from social media played a critical role in this process; as individuals understood market dynamics, they tended to control their emotional responses, develop patience, and make more rational investment decisions. Indeed, studies have shown that as access to information and financial literacy increase, individuals’ ability to cope with stress also strengthens (Kalfa and Yılmaz, 2024). Conversely, the absence of social support often intensified feelings of isolation, emotional burden, and psychological distress. These findings further support Stress and Coping Theory by demonstrating the importance of social resources in managing uncertainty and maintaining psychological well-being (Lazarus and Folkman, 1984; Thoits, 2011).

*“As these losses increased, I found myself unable to tell anyone about the situation, and this began to make me feel indifferent. In fact, I lost approximately $15,000 within a year and started hiding this from my family so they wouldn’t find out. I tried to deal with all this negative burden on my own, and it started to wear me down. However, at times when I started making profits, I felt a brief sense of relief from the stress.” (K12: 4B1, 4B2, 4B3)*

*“Actually, at first, I made purchases based entirely on intuition and the desire to make a lot of money. But now I’ve started to proceed in a more planned and analytical manner. Actually, my friends and cousin guided me in my first investment, but later I made my own decisions. However, when I lost all my money, I felt lonely and couldn’t share it with my family. Yes, they didn’t know, but if they had, I’m sure I would have experienced negative things because I was still buying and selling.” (K13: 4B1, 4B2, 4B3)*

Taken together, Theme 4 illustrates that coping with uncertainty was shaped not only by individual efforts but also by the quality of participants’ social relationships. Experiences of support, trust, isolation, and resilience extended beyond immediate emotional adjustment and gradually influenced how participants perceived themselves as investors. As these experiences accumulated over time, cryptocurrency investment became increasingly integrated into participants’ broader self-concepts and personal value systems. This progression provides the foundation for the final theme, which examines identity transformation and the development of self-awareness through lived investment experiences.

### Theme 5. Identity dilemma and transformation in self-awareness sub-theme 1. Investor identity and internal conflicts

The cryptocurrency investment process has become not only a financial practice for individuals but also an important component of identity construction. Over time, the tendency to identify with the investor role strengthened considerably. While financial success often contributed to increased self-confidence and a stronger sense of competence, losses and uncertainty frequently intensified the need for control and self-evaluation. This situation created internal tensions for many participants. On the one hand, investors sought to develop disciplined, analytical, and rational investment strategies; on the other hand, they continued to experience emotionally driven reactions to gains, losses, and market volatility. Similar tensions between emotional responses and financially oriented self-concepts have been discussed in studies examining investor identity and financial decision-making (D’Acunto, 2015; Monk and Rook, 2023). Consistent with these findings, participants in the present study frequently described an ongoing negotiation between emotional impulses and the desire to maintain a disciplined investor identity. As a result, they often reflected on whether their increasing commitment to cryptocurrency investment had begun to influence other aspects of their personal lives and self-perceptions. As a result, participants often described a conflict between their emotional self and their emerging investor identity, raising questions such as whether increasing involvement in cryptocurrency investment had begun to overshadow other aspects of their lives and personal identities. These findings are consistent with Social Identity Theory, which suggests that individuals derive part of their self-concept from meaningful social roles and group memberships (Tajfel and Turner, 1979; Haslam, 2004). Overall, these findings suggest that cryptocurrency investment gradually became integrated into participants’ broader sense of self rather than remaining a purely financial activity. Emotional experiences, repeated adaptation to uncertainty, and social interactions collectively contributed to changes in self-perception, personal priorities, and long-term identity development (Tajfel and Turner, 1979; Haslam, 2004). The following narratives demonstrate how participants described this evolving process of identity transformation through their lived experiences.

*“So, first of all, it taught me that I shouldn’t invest in something I don’t know enough about without doing my research and educating myself, that I need to learn how to achieve financial success as a rational investor, and how to cope with the emotional void that arises when I suffer a loss. I developed myself in terms of significant behavioral changes between my previous self and my current self, personal empowerment, coping with feelings of exhaustion, etc. But I can say that I also lost my mental health.” (K12: 5A1, 5A2, 5A4, 5A6, 5A8)*

*The cryptocurrency investment process taught me once again how valuable patience is. As a result of this experience, the happiness and self-confidence brought about by financial success began to turn into unease and unhappiness due to my early selling of investments in later periods. After seeing what my investment over four years could change in my life, I learned that investing is not about buying and selling, but about buying and waiting. I learned that, instead of momentary panic and instant transactions, a good investment made with patience and over the long term can greatly change a person’s life. During this period, my behavior was characterized by excitement, momentary anxiety, or fear, but my experiences led me to the idea that patiently waiting for well-planned and strong projects to invest in would lead to great financial success.” (K14: 5A1, 5A2, 5A3, 5A8)*

*“Cryptocurrency investment was like an accelerated course in finance, psychology, and patience. It taught me that wealth isn’t built overnight and that emotions can be as dangerous as market crashes. Initially, I was chasing quick riches, but losses showed me the value of discipline. For example, during an Ethereum drop in 2023, I held on instead of selling and made a 40% profit in 2024. This taught me to trust the fundamentals. The biggest lesson was self-awareness. Crypto brought out my impulsive side; I was making instant coin purchases driven by FOMO. Recognizing this flaw led me to set rules like a 48-h waiting period. This discipline has spilled over into my life: I am now more conscious about budgeting and career planning. Crypto also taught me resilience. The most significant change was my perspective on risk. I used to avoid uncertainty; I was tied to a secure 9-to-5 job and a savings account. Crypto showed me that calculated risks can pay off. This gave me the courage to take on part-time freelance work; I diversified my income like my portfolio. Failure no longer paralyzes me; instead, I view mistakes as data. Socially, I learned to filter advice. My early addiction to the hype on X proved costly, but now I prioritize quality sources over noise. This critical thinking helps me sift through the noise in my life, from career advice to news. Crypto didn’t just change my wallet; it reshaped my approach to decision-making, risk, and growth.” (K1: 5A1, 5A2, 5A3, 5A5, 5A7, 5A8)*

### Sub-theme 2. Life priorities

The cryptocurrency investment process stands out as an experiential field that reshapes individuals’ life priorities and leads to significant changes in their behavior patterns. Although negative emotions such as impatience, exhaustion, and loss of self-confidence are commonly observed in the early stages, over time, the importance of patience and strategy is understood, increasing investors’ self-awareness. OECD reports indicate that in an environment of increased financial literacy, individuals establish more sustainable priorities in both their investment decisions and lifestyles (OECD/INFE, 2018). The stress and loneliness caused by losses wear individuals down psychologically due to the inability to share the emotional burden, while strategic approaches (e.g., setting specific goals, long-term planning) and support systems help to rearrange life priorities. However, investors’ resilience alters the priority order of their value judgements regarding other aspects of life through risk tolerance and financial stress management; over time, investors come to value not only “making a profit” but also psychological balance, having time for themselves, maintaining social relationships, and inner peace. Ultimately, the investment experience evolves away from the pursuit of short-term gains toward a long-term identity transformation, leaving lasting effects on both individuals’ financial decisions and lifestyles. This transformation is closely related not only to financial decision-making processes but also to concepts such as psychological well-being, opportunity perception, and life satisfaction (Liu et al., 2022). The transformation of life priorities reported by participants further suggests that cryptocurrency investment may extend beyond financial decision-making and contribute to broader processes of self-awareness, identity development, and personal meaning-making. These findings support recent research emphasizing the role of identity-related processes in shaping long-term behavioral patterns and life choices (Stets and Burke, 2000; Haslam, 2004).

*“The greatest experience this process has given me is doing something I didn’t know how to do. I came to believe that the most important feature of the stock market world is patience, and that impatient, uninformed, inexperienced people should not invest here. During this process, I realized that making money and turning a profit is very difficult, and that this business cannot be done with hearsay and luck. The feelings of exhaustion, loneliness, stress, and loss of self-confidence I experienced in the early days of entering this market with big dreams eventually led to positive changes in my financial success and behavior.” (K13: 5B1, 5B2, 5B3, 5B4)*

*“The cryptocurrency investment process taught me once again how valuable patience is. As a result of this experience, the happiness and self-confidence brought by financial success began to turn into unease and unhappiness over having sold my investments too early in later periods. After seeing what my investment over four years could change in my life, I learned that investing is not about buying and selling, but about buying and holding. I learned that a well-planned, patient, and long-term investment can profoundly change a person’s life, rather than acting on momentary panic or making impulsive trades. During this period, my behavior was characterized by excitement, momentary anxiety or fear, but my experiences led me to the idea that patiently waiting for well-planned and strong projects to invest in would lead to great financial success.” (K14: 5B1, 5B2, 5B4)*

*“Personally, I understood the importance of being patient and staying calm in the face of sudden changes during this process. It is a stressful process. Moreover, these endeavors put your professional career at risk because your work suffers as you chase after cryptocurrency. From this perspective, I also think it harmed my professional life for several years; for example, I didn’t read a single book, I spent my nights staring at the screen watching coins. Moreover, I suffered financial losses due to sudden sales I made or, sometimes, due to sales I didn’t make during days of decline. If you’re in this market, you need to stay calm, be resilient in the face of stress, and occasionally take a break by shifting your focus and forgetting about your money for a while. But unfortunately, especially in our country where money is the goal, it seems very common to tie up what little money you have here and then easily lose it all. I genuinely feel sorry when I see people around me taking out loans or selling their homes and cars, believing they can get rich quickly, and investing everything into cryptocurrency. The stock market isn’t like that, though; it requires patience, a bit of strategy, and experience. We take it too lightly and dive in. I’ve even heard some people use the word “play.” I’ve wondered what they think they’re playing at in the crypto market. It’s so easy to win, but in the end, you see them crying because they’ve lost everything. That much stress was too much for me, of course. At the end of the process, even though I had acquired a good amount of Eth, I said, “Enough is enough,” because my blood pressure was going up and down.” (K18: 5B1, 5B2, 5B3, 5B4)*

## Discussion

### Cryptocurrency investment as a socio-psychological adaptation process

The findings of the present study suggest that cryptocurrency investment is more appropriately understood as a continuous socio-psychological adaptation process than as a sequence of isolated financial decisions or discrete behavioral responses. Rather than experiencing emotional regulation, social influence, coping, and identity construction as separate phenomena, participants described these processes as dynamically interconnected throughout their investment journeys. Their lived experiences indicate that emotional reactions to market volatility initiated an adaptive process in which repeated encounters with uncertainty gradually reshaped decision-making practices, patterns of social interaction, and personal identity. This integrated pattern extends beyond conventional behavioral explanations that typically examine emotional biases, social influence, or risk perception as independent determinants of investment behavior (Kahneman and Tversky, 1979; Statman, 2019).

From a phenomenological perspective, this interconnectedness represents an important analytical contribution. Participants did not describe their experiences as isolated moments of fear, confidence, or social influence. Instead, they portrayed investment as an evolving process in which emotional responses, coping efforts, interpersonal relationships, and self-perceptions continuously influenced one another. Emotional regulation facilitated more deliberate decision-making; repeated coping with uncertainty strengthened confidence and behavioral discipline; social interactions simultaneously provided informational resources and emotional reassurance; and these accumulated experiences gradually became incorporated into participants’ identities as investors. Consequently, the findings suggest that cryptocurrency investment should be conceptualized not simply as financial behavior occurring within uncertain markets but as an evolving socio-psychological process of adaptation through which individuals continuously reconstruct both their investment practices and their understanding of themselves (Gross, 1998; Lazarus and Folkman, 1984; Tajfel and Turner, 1979).

Importantly, this interpretation also helps explain why participants’ experiences frequently transcended the behavioral patterns commonly emphasized within the cryptocurrency literature. While previous studies often focus on impulsivity, herd behavior, overconfidence, or emotional decision-making (Lo, 2017; Sudzina et al., 2021; Jain et al., 2025), the present findings indicate that these responses were neither fixed nor universal. Instead, participants frequently described progressive learning, increasing self-regulation, and behavioral adaptation as they accumulated investment experience. This suggests that investor behavior is not adequately explained solely by the presence of behavioral biases; rather, these biases may themselves evolve through ongoing emotional, cognitive, and social adaptation. Viewing cryptocurrency investment through this broader socio-psychological lens therefore provides a more dynamic understanding of investors’ lived experiences than approaches that conceptualize investment behavior primarily as the outcome of isolated psychological mechanisms.

### From emotional regulation to identity transformation: an integrated process

One of the principal contributions of the present study lies in demonstrating that emotional regulation, coping, social interaction, and identity construction are not independent dimensions of cryptocurrency investment but successive and mutually reinforcing stages of a broader adaptation process. Although Emotion Regulation Theory, Stress and Coping Theory, and Social Identity Theory have generally been applied as separate explanatory frameworks, the lived experiences reported by participants indicate that these processes unfolded in a continuous and interconnected manner. Emotional regulation represented the initial response to market volatility, but this alone did not sustain long-term investment engagement. Instead, participants gradually developed coping strategies that reduced emotional distress, encouraged more deliberate decision-making, and strengthened psychological resilience under persistent uncertainty (Gross, 1998; Lazarus and Folkman, 1984; Folkman and Moskowitz, 2004).

Importantly, these adaptive processes did not remain confined to individual psychological regulation. As participants accumulated investment experience, interactions with family members, friends, online communities, and digital financial platforms increasingly shaped how they interpreted market events, evaluated risks, and assessed their own investment competence. Social interaction therefore functioned not merely as an external source of information but as an active component of the adaptation process itself. Through repeated participation in these social environments, participants gradually reconstructed their understanding of themselves as investors, suggesting that identity development emerged from the cumulative interaction of emotional, behavioral, and social experiences rather than from social belonging alone (Tajfel and Turner, 1979; Haslam, 2004; Papadamou et al., 2023).

This integrated interpretation also refines existing behavioral finance perspectives. Much of the behavioral finance literature explains cryptocurrency investment by emphasizing cognitive biases, emotional reactions, and heuristic decision-making (Kahneman and Tversky, 1979; Statman, 2019). While these mechanisms remain important, the present findings suggest that they represent only particular stages within a broader developmental process. Participants frequently described how behaviors initially characterized by uncertainty, emotional volatility, or social dependence gradually evolved into more reflective, self-regulated, and strategically organized investment practices. Consequently, behavioral responses should not necessarily be viewed as stable characteristics of cryptocurrency investors but as dynamic experiences that may change through learning, repeated exposure to uncertainty, and continued social participation. This perspective complements rather than contradicts existing behavioral finance theories by highlighting the temporal and experiential processes through which investor behavior develops. Taken together, these findings indicate that cryptocurrency investment is best understood as an evolving socio-psychological process in which emotional regulation facilitates coping, coping is reinforced through social interaction, and repeated social experiences gradually contribute to identity formation. Viewing these dimensions as sequential yet mutually reinforcing processes provides a more integrated explanation of investors’ lived experiences than approaches that examine emotional, cognitive, or social mechanisms in isolation.

### The distinctive contribution of a phenomenological perspective

While previous research has substantially advanced understanding of cryptocurrency investment through quantitative analyses of behavioral biases, risk perception, emotional reactions, and market behavior, these approaches generally explain investment decisions by examining relationships between predefined psychological variables (Kahneman and Tversky, 1979; Lo, 2017; Statman, 2019). Such studies have provided valuable evidence regarding factors associated with investment behavior but have offered relatively limited insight into how these psychological and social processes are experienced, interpreted, and reconstructed throughout investors’ everyday lives.

The phenomenological approach adopted in the present study contributes a different perspective by focusing on the lived experience of cryptocurrency investment rather than on isolated behavioral outcomes. Instead of treating emotional regulation, coping, social influence, or identity as independent variables, participants described these dimensions as continuously intertwined throughout their investment journeys. Emotional reactions influenced subsequent coping strategies; coping experiences shaped interactions with other investors; social relationships affected confidence, belonging, and decision-making; and these accumulated experiences gradually contributed to changes in identity, self-awareness, and personal priorities. Consequently, the findings suggest that cryptocurrency investment is not simply characterized by behavioral biases or emotional responses but represents an evolving process through which individuals continuously reinterpret both uncertain market environments and their own place within them.

Importantly, this perspective also complicates some common assumptions within the cryptocurrency literature. Existing research frequently portrays cryptocurrency investors as highly impulsive, emotionally reactive, and particularly susceptible to cognitive biases, herding behavior, and speculative decision-making (Sudzina et al., 2021; Bland et al., 2024; Jain et al., 2025). Although such behaviors were evident in participants’ accounts, they did not remain stable over time. Instead, many participants described gradual transitions toward greater emotional awareness, more structured decision-making, increased psychological resilience, and stronger confidence in managing uncertainty. These findings therefore suggest that behavioral biases should not necessarily be interpreted as permanent characteristics of cryptocurrency investors but may instead represent transitional experiences within broader processes of adaptation and learning.

From this perspective, the contribution of the present study extends beyond identifying individual psychological or social factors associated with cryptocurrency investment. Rather, it demonstrates how these factors become interconnected through lived experience and gradually evolve into a dynamic process of socio-psychological adaptation. This interpretation complements existing behavioral finance research by revealing not only what influences cryptocurrency investment behavior but also how these influences develop, interact, and acquire personal meaning throughout investors’ experiences. In doing so, the study illustrates the distinctive value of phenomenological inquiry for understanding cryptocurrency investment as a continuously evolving human experience rather than merely a financial decision-making activity. Accordingly, the present findings encourage a shift from viewing cryptocurrency investment primarily as a set of behavioral outcomes toward understanding it as an evolving socio-psychological adaptation process in which emotions, coping practices, social relationships, and identity continuously shape one another through lived experience.

### Theoretical and practical implications

The present findings have several theoretical implications for behavioral finance and cryptocurrency research. First, the study suggests that cryptocurrency investment should not be conceptualized solely through isolated behavioral biases or discrete psychological constructs. Instead, investors’ experiences indicate that emotional regulation, coping strategies, social interaction, and identity construction operate as interconnected processes that evolve throughout continued market participation. This integrated perspective complements existing behavioral finance theories by emphasizing the developmental and experiential nature of investment behavior rather than viewing behavioral responses as fixed individual characteristics (Kahneman and Tversky, 1979; Statman, 2019). Second, the findings contribute to the growing literature on cryptocurrency investment by demonstrating that adaptation to uncertainty involves more than improving financial decision-making skills. Participants described learning to regulate emotions, evaluate information more critically, seek appropriate social support, and gradually redefine their identities as investors. These observations suggest that long-term engagement in cryptocurrency markets is influenced not only by financial knowledge but also by broader socio-psychological adaptation processes. Consequently, future research may benefit from examining cryptocurrency investment through longitudinal and process-oriented perspectives capable of capturing how investor experiences change over time rather than focusing exclusively on cross-sectional behavioral outcomes.

The findings also have practical implications. Financial education programmes designed for cryptocurrency investors should extend beyond traditional concepts such as portfolio diversification and risk management by incorporating emotional regulation, uncertainty management, critical evaluation of online financial information, and awareness of social influence. Given the increasing role of digital communities and finfluencers in shaping investment behavior, educational initiatives should also encourage investors to critically evaluate online financial content and recognize both the potential benefits and risks associated with socially mediated investment decisions (Hayes and Ben-Shmuel, 2024; Papadamou et al., 2023).

Finally, the present study highlights the value of phenomenological inquiry for advancing research on cryptocurrency investment. Rather than identifying only the psychological or social factors associated with investment behavior, the findings demonstrate how these dimensions become progressively interconnected throughout investors’ lived experiences. Accordingly, the study proposes that cryptocurrency investment is most appropriately understood as a dynamic socio-psychological adaptation process through which individuals continuously regulate emotions, develop coping strategies, negotiate social relationships, and reconstruct their identities while engaging with uncertain financial environments. This perspective not only enriches existing behavioral finance research but also provides a more comprehensive understanding of cryptocurrency investment as a lived human experience. Rather than portraying cryptocurrency investors as individuals who merely exhibit behavioral biases, the present study demonstrates that they actively adapt to uncertainty by continuously reconstructing emotional regulation, coping practices, social relationships, and identity through lived experience.

## Conclusion and recommendations

This study suggests that cryptocurrency investment is best understood as an evolving socio-psychological adaptation process rather than a sequence of isolated financial decisions or behavioral responses. Participants’ lived experiences revealed that emotional regulation, coping strategies, social interactions, and identity construction developed as dynamically interconnected processes that continuously shaped one another throughout their investment journeys. The findings reveal that investors typically begin their investment journeys with excitement, curiosity, and high expectations, yet prolonged exposure to market volatility gradually transforms these initial reactions into more disciplined, reflective, and strategic forms of behavior. Over time, participants reported greater emotional awareness, improved risk evaluation, stronger self-regulation, and more adaptive coping strategies in response to uncertainty and financial stress. These findings indicate that investors’ behavioral responses were not static but progressively evolved through repeated engagement with uncertainty, learning, and social experience. Participants’ experiences further indicate that cryptocurrency investment is embedded within broader social environments that influence motivation, decision-making, and identity development. Family members, friends, social media content, online communities, and digital financial influencers emerged as important sources of information, encouragement, and social validation. However, as investors gained experience, they increasingly shifted from externally guided decisions toward independent analysis, critical evaluation, and rule-based investment practices. At the same time, investment experiences contributed to the development of investor identity, shaping self-perceptions, life priorities, and long-term behavioral patterns. From a theoretical perspective, the study contributes to the behavioral finance literature by demonstrating that cryptocurrency investment cannot be fully understood through financial outcomes and cognitive biases alone. Instead, investors’ experiences are shaped by the interaction of emotional regulation, coping mechanisms, social influences, community attachment, and identity-related processes. Rather than applying Emotion Regulation Theory, Stress and Coping Theory, and Social Identity Theory as separate explanatory perspectives, the present study demonstrates that their integration provides a more comprehensive explanation of how investors psychologically and socially adapt to persistent uncertainty in cryptocurrency markets. This integrated perspective extends existing behavioral finance research by emphasizing adaptation as a dynamic and evolving process rather than a collection of isolated behavioral mechanisms. From a practical perspective, investor education initiatives should extend beyond technical market knowledge and incorporate behavioral, emotional, and psychological dimensions of investing. Training programmes focusing on emotional self-regulation, critical evaluation of online information, responsible risk management, and awareness of social influence may contribute to healthier investment decisions and more sustainable participation in cryptocurrency markets. Overall, the present study encourages a shift from viewing cryptocurrency investment primarily through behavioral biases and financial outcomes toward understanding it as an evolving socio-psychological adaptation process through which individuals continuously reconstruct emotional regulation, coping practices, social relationships, and identity through lived experience.

### Limitations and future research

Several limitations of this study should be acknowledged. First, the findings are based on a relatively small sample of cryptocurrency investors from a specific regional context in Türkiye, which may limit the transferability of the findings to other populations and cultural settings. Second, the cross-sectional nature of the study captures participants’ experiences at a particular point in time and does not allow direct observation of how emotional responses, coping processes, and identity-related experiences evolve over time. In addition, the sample was predominantly male, with only two female participants included in the study. Although previous studies have reported higher levels of male participation in cryptocurrency markets, the limited representation of female investors may have restricted the diversity of perspectives captured. Furthermore, because the findings were derived from retrospective accounts of past investment experiences, they may be affected by recall bias and *post hoc* rationalization. Participants’ recollections of emotions, decisions, and reactions may not fully reflect their perceptions at the time the events occurred, and some experiences may have been reinterpreted in light of subsequent outcomes and accumulated investment experience. Future research may employ longitudinal and mixed-method designs to examine changes in investor experiences across different stages of cryptocurrency investment. Comparative studies involving diverse cultural contexts, demographic groups, and market conditions may also contribute to a more comprehensive understanding of the psychological and social dimensions of cryptocurrency investment.

## Data Availability

The data supporting this study originate from qualitative interviews conducted with 19 participants. To preserve participant confidentiality, these data are not publicly available. However, anonymized interview materials can be accessed from the corresponding author upon reasonable request. Requests to access these datasets should be directed to AB, kadirbarut@harran.edu.tr.

## Appendix 1 | COREQ checklist.

**TABLE A1:**

Area	Number	Item	Guiding questions	Explanations
**Area 1: Research** **Team and Reflexivity**	1	Interviewer/Facilitator	Who conducted the interviews with the participants?	All interviews with participants were conducted personally by the first author of the study.
Personal Characteristics	2	Identity Information	What academic degrees or professional titles does the researcher hold?	The corresponding author holds a doctoral degree.
	3	Occupation	What role or professional position did the researcher hold during the study?	The researcher holds a doctoral degree and serves as an Assistant Professor in the field of finance and behavioral finance.
	4	Gender	What is the gender of the researcher conducting the interviews?	The researcher who conducted the interviews is male.
	5	Experience and	What is the	The researcher has
		Education	researcher’s	extensive academic
			educational	knowledge and practical
			background and areas	experience in qualitative
			of expertise?	research methods.
**Relationship with** **Participants**	6	Relationship Status	Was there any contact or acquaintance between the researcher and the participants prior to the start of the research?	No personal contact was established with participants prior to the research. Participants were only informed about the researcher’s academic identity and the purpose of the study.
	7	Participants’ Knowledge About the Researcher	What information did the participants have about the researcher conducting the interview?	Participants were informed that the researcher conducting the interviews held a doctoral degree and worked at an academic institution.
	8	Interviewer Characteristics	Were the researcher’s personal perspectives, biases, or motivations for the research disclosed?	Before the commencement of data collection and analysis, the researcher critically reflected on personal assumptions and potential preconceptions regarding cryptocurrency investment behavior. Throughout the research process, a reflexive approach was maintained to minimize the influence of prior beliefs on data interpretation. During interviews, participants were encouraged to describe their experiences freely, and only non-directive follow-up questions were used to clarify meanings and encourage elaboration. In the analysis stage, interpretations and emerging themes were periodically reviewed with an independent academic expert to reduce subjective bias and strengthen analytical credibility. Participants were informed only about the researcher’s academic background and the purpose of the study.
**Area 2: Research Design – Theoretical Framework**	9	Theoretical Framework	What theoretical or methodological framework guided the study?	The study was grounded in a phenomenological research framework, and interpretive techniques were used throughout the process.
**Participant Selection**	10	Sampling	How were the individuals participating in the study selected and according to which criteria?	Participants were selected using purposive sampling, and a criterion sampling strategy was preferred in this context. Criteria: (a) having at least 12 months of experience in cryptocurrency investment, (b) volunteering to participate, (c) being able to express oneself in Turkish.
	11	Access Method	How were participants invited or accessed for the study?	Participants were recruited through purposive and referral-based sampling. Initial participants were identified through the researcher’s professional and social networks among individuals known to have experience in cryptocurrency investment. Subsequently, several participants referred other investors who met the inclusion criteria. Interviews were arranged according to participants’ availability and willingness to participate.
	12	Sample Size	How many participants were included in the study?	A total of 19 people were included in the study.
	13	Non-participants	Were there any individuals among those invited who declined to participate or were not included in the study?	All individuals invited to participate in the study agreed to participate.
**Research Environment**	14	Data Collection Environment	Where or in what settings were the interviews and data collection conducted?	The data collection process was conducted in locations deemed appropriate by the participants (e.g., a quiet office environment or a suitable room at home).
	15	Others Present	Were there any other individuals present during the interview besides the researcher and the participant?	The interviews were conducted one-on-one between the researcher and the participant only.
	16	Definition of the Sample	What are the basic demographic and professional characteristics of the participant group?	The participants’ age, gender, education and occupation information is presented in a summary table.
**Data Collection**	17	Interview Guide	How were the interview questions developed and finalized?	The interview questions were prepared based on a literature review, reviewed by experts, and finalized after a pilot test.
	18	Follow-up Interviews	Were multiple interviews conducted with participants?	Only one interview was conducted with each participant; no repeat interviews were conducted.
	19	Recording	Was the interview process recorded, and how was it recorded?	The interviews were recorded using audio recording.
	20	Field Notes	Did the researcher record their observations or additional notes during the interview?	The participants’ statements and the researcher’s observations were noted in detail.
	21	Duration	What was the average duration of the interviews?	The duration of the interviews ranged from 30 to 50 min.
	22	Data Saturation	At what stage was data saturation achieved in the study?	Data saturation was reached after the fifteenth interview, as no substantially new themes or meanings emerged from subsequent interviews. Nevertheless, four additional interviews were conducted to confirm thematic stability and strengthen the robustness of the findings.
	23	Sharing of Transcripts	Were the transcripts of the interview recordings sent to the participants for review?	The interview transcripts were not returned to participants for review. Given the sensitive nature of financial experiences and personal investment decisions, the researchers sought to minimize potential privacy concerns and participant burden. Furthermore, the study prioritized maintaining participants’ comfort and confidentiality throughout the research process.
**Field 3: Analysis and Findings**	24	Number of Coders	Who conducted the analysis of the data?	The primary stages of data collection, coding, and analysis were conducted by the first researcher. The resulting codes, sub-themes, and themes were subsequently reviewed by a second academic expert to enhance analytical credibility and interpretive consistency.
	25	Coding Tree	Was the structure or scheme used in the coding process	The coding process was transparently structured in the form of raw data,
			explained in detail?	meaning units, codes, and sub-themes. A sample table related to the coding tree is presented in the findings section.
	26	Derivation of Themes	How were the themes determined in the study, and through which stages?	Themes were derived from the experiences shared by participants. To increase reliability, after the coding process was completed, the findings were compared with a second field expert and consensus was reached.
	27	Software	Was any software programme used in the data analysis process?	Data analysis was conducted directly by the researcher without the use of any software.
	28	Participant Verification	Did the participants review and approve the findings?	No further contact was made with the participants after the study was completed.
Reporting	29	Quotations	Were the participants’ direct statements included in the presentation of the findings?	The findings are supported by quotations from the participants’ direct statements.
	30	Consistency	Was consistency achieved between the collected data and the findings obtained?	The findings reached in the research were presented in a manner consistent with the collected data.
	31	Clarity of Main Themes	Have the main themes been presented in a clear and understandable manner in the study?	The main themes were presented clearly in the study.
	32	Clarity of Sub-Themes	Have the sub-themes been defined in detail and clarified?	The sub-themes have been addressed and explained in detail.
